# Predicting Rural Women's Breast Cancer Screening Intention in China: A PLS-SEM Approach Based on the Theory of Planned Behavior

**DOI:** 10.3389/fpubh.2022.858788

**Published:** 2022-04-11

**Authors:** Yanjun Sun, Jiawei Yuan, Wuqianhui Liu, Banghui Qin, Zhiqing Hu, Jianwei Li, Yuan He

**Affiliations:** ^1^Institute of Medical Humanities, Nanjing Medical University, Nanjing, China; ^2^School of Marxism, Nanjing Medical University, Nanjing, China; ^3^School of Health Policy and Management, Nanjing Medical University, Nanjing, China; ^4^The First School of Clinical Medicine, Nanjing Medical University, Nanjing, China; ^5^Department of Material and Child Health, Lianyungang Material and Child Health Hospital, Lianyungang, China; ^6^Research Center for Social Risk Management of Major Public Health Events (Key Research Base of Philosophy and Social Sciences of Universities in Jiangsu), Nanjing Medical University, Nanjing, China

**Keywords:** breast cancer screening intention (BCS intention), rural women, the theory of planned behavior (TPB), the partial least square structural equation model (PLS-SEM), multi-group analysis (MGA)

## Abstract

**Background:**

It was reported that the incidence of breast cancer (BC) was the highest among cancers worldwide. The breast cancer screening (BCS) program is regarded as an effective preventive measure. However, rural women's willingness to participate in the BCS program is relatively low. To provide measures to prevent BC, it is necessary for the government to identify the influencing factors of rural women's BCS intention.

**Methods:**

A cross-sectional study was conducted among 3,011 rural women by a convenience sampling method through face-to-face interviews on a self-designed questionnaire based on the theory of planned behavior (TPB). The partial least square structural equation model (PLS-SEM) was conducted to determine the predictors of BCS intention, and a multi-group analysis (MGA) of age was performed to identify if there were differences in all hypotheses between different age groups.

**Results:**

There were still rural women who have not been screened for BC in five years (41.7%). The research model of rural women's intention to accept this prevention against BC was rational. All of the hypotheses are supported. Especially, subjective norm (SN) (β = 0.345, *p* < 0.001) is found to be the strongest predictor followed by the perceived behavioral control 1 (PBC 1) (personal factors, including distance, transportation, busyness, etc.) (β = 0.165, *p* < 0.001), attitude (β = 0.152, *p* < 0.001), past behavior (PB) (β = 0.150, *p* < 0.001), knowledge (β = 0.121, *p* < 0.001), and perceived behavioral control 2 (PBC 2) (pain and cultural-social factors including embarrassment from a physician, etc.) (β = 0.042, *p* < 0.05). The advocacy and education (A&E), medical level and service attitude (ML&SA) of township health centers and village clinics can affect behavior intention (BI) *via* attitude, SN, and PBC. The results of MGA of age indicate that there are significant differences among rural women of different ages regarding the relationship between A&E and PBC 2 (*p* < 0.01) and the effect of PB on BI (*p* < 0.001).

**Conclusion:**

The TPB with the addition of PB, knowledge, ML&SA, and A&E can provide the theoretical basis for the policy intervention that aims to enhance the rural women's BCS willingness. MGA of age is conducive to promoting the implementation of the BCS policy. The findings are of great significance to improve rural women's health levels.

## Introduction

The previous studies suggested that breast cancer's incidence and mortality in developed countries have decreased obviously in recent years, while the prevalence in developing countries has increased gradually (1). According to the estimates of the International Agency for Research on Cancer (IARC) on the global burden of cancer in 2020, female breast cancer (BC) was estimated to be the top of the 10 most common cancer types (2). It was considered that female breast cancer was the most commonly diagnosed cancer worldwide, which accounted for 11.7% of the total newly diagnosed cancer cases. It also showed 6.9% of the total cancer deaths, which ranked fifth (2). The statistics from the National Cancer Registry showed that the incidence of breast cancer among rural women was 79 per 100,000 in 2015 (3). The statistics from the National Health and Family Planning Commission of PRC showed that the death rate reached 6.48 per 100,000, ranking the 4th highest incidence of all cancers among rural women in China (4). The past study also demonstrated that poor women were more likely to develop BC than those with higher family income and urban residence due to the limited detection and screening facilities as well as fewer opportunities to seek better medical treatment (5). It appears that breast cancer has been a major public health problem globally, especially among the rural women who deserve more attention.

However, the etiology of breast cancer is unclear now (6, 7). But a lot of studies have confirmed that early diagnosis and treatment can reduce mortality significantly (7), and screening services play a significant role in improving the early diagnosis rate (8). According to studies in developed countries, high coverage of breast cancer screening (BCS) can effectively reduce mortality. For example, BCS was national coverage in the United Kingdom in the mid-1990s with women over 50 using breast Xray every 3 years. Thus, the mortality among patients with breast cancer aged 55~69 decreased by 1/3 (7). In the United States, Australia, et al., the BCS program has been a national policy and continues to be promoted (7). The World Health Organization (WHO), International Union Against Cancer (UICC), and the American Cancer Society (ACS) have concluded that the BCS program is effective and is worth promoting worldwide (7).

In China, BC was considered to be one of the leading malignant tumors and the main cause of cancer death in women below 45 years old in 2015 (3), and there was also an increasingly upward trend in the rates of age-standardized incidence and mortality (3). Researchers predicted that there would be 2.5 million women aged 45–59 with BC by 2021 (9). The Chinese government always attaches great importance to BCS, and rural women's BCS has been included in the major public health services since 2009 (10). Unfortunately, even with free screening services, rural women still lacked willingness to be engaged in the screening, and the screening rate was not high (11). It was demonstrated that the rate in China rural was lower than it was in urban and far lower than it was in the developed countries. For example, the BCS rate for rural of Jilin Province only reached 9.09% in 2013 (12). Even in economically developed regions, the screening rate was not satisfying either, only reaching 38.09% of Conghua District, Guangzhou (13) and 23.3% of Wenling, Zhejiang in 2015 (14). The past research revealed that the screening rate was 38.05% in rural, while 48.09% in urban (15). As for developed countries, the BCS rate was 72.4% in the United States in 2010 and more than 70% rural women have done a screening for breast cancer and cervical cancer within 5 years in the Netherlands (16). Hence, the enthusiasm of Chinese rural women to undergo BCS urgently needs improvement.

In order to improve Chinese rural women's screening participation, the influencing factors of their BCS intention should be emphasized when designing and implementing the BCS program. However, few studies specifically focused on rural women's intention to BCS in China. Only a small number of studies examined the factors that influence BCS behavior based on socio-demographic characteristics, which are the education level, monthly income, age, etc. (17). Most studies in China did not draw on social psychological theories or behavioral theories. Whereas, with the deepening of the research, academic community has come to realize that screening is a healthy behavior that requires long-term persistence and is affected by multiple factors of the physical and social environment (7). Therefore, it is urgent to conduct empirical studies to explain and predict individual behavior of rural women's BCS in China. Subsequently, there were some studies that investigated the personal health beliefs (18) and external environmental factors (19, 20), such as the society or organizations. These studies were generally based on social psychological models, including health belief model (HBM) (18, 21) and the theory of rational behavior (TRA) (21). But the views on health HBM emphasize more about the influence of individual cognition on health behavior and consider less about the social factors. The theory of planned behavior (TPB) incorporates perceived behavioral control on the basis of TRA (22). The structural model of TPB can measure not only the internal factors but also the characterization of the social environment, and it has been proved to effectively explain and predict the health prevention behavior and behavior intention (23). It is widely used in the field of health prevention behavior, including AIDS prevention (24), smoking interventions (25), cervical cancer screening (26), etc. However, only a few pieces of research evaluated breast self-examination and its effective factors (27) and the role of educational intervention in mammography screening based on TPB (28). Fewer pieces of research evaluated the rural women's breast screening intention based on TPB.

Therefore, using a PLS-SEM approach based on the TPB, this study aimed to predict the women's BCS intention and to analyze its influencing factors in rural China in order to promote women's health, and further research in this area from rural women's perspective is needed.

**Figure 1:**
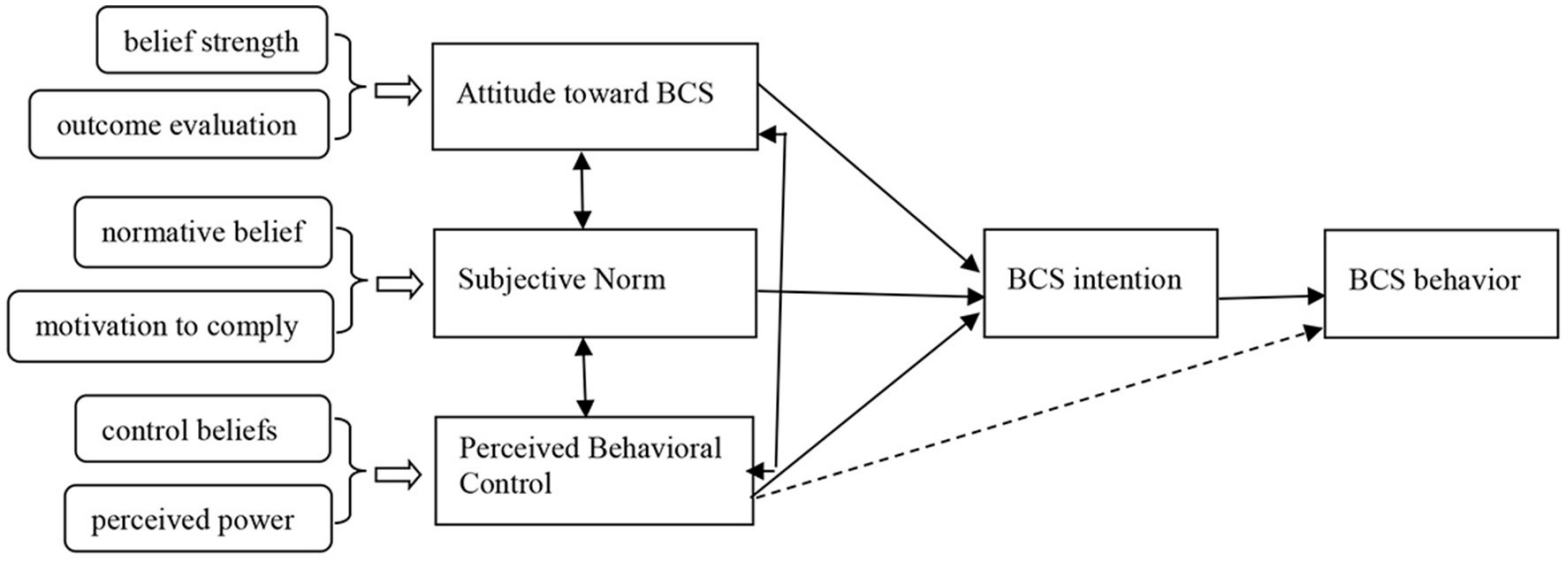
A structural model of the theory of planned behavior (31).

Theory of planned behavior is a social cognitive theory that explains how attitude toward the behavior (AB), subjective norm (SN), and perceived behavioral control (PBC) act on behavior intention (BI) and then on actual behavior as shown in Figure 1 (29). In this model, attitude, SN, and PBC are independent and pairwise. Accurate PBC can be used as an alternative measure of actual control conditions to directly predict the possibility of behavior occurrence (as illustrated in the dashed line in Figure 1) (29). “Attitude toward the behavior (AB) refers to a person's general and stable tendency to perform a certain behavior (29). The tendency often contains two separable components: belief strength (b) and outcome evaluation (e), as shown in the Equation (1) (22, 30) “AB ∝ ∑*b*_*iei*_.”

(*i means measurement project*). SN is defined as individuals' beliefs on the extent to which others would expect them to perform a behavior (29). The measurement of SN also contains two separable components, normative belief (n) and motivation to comply (m), as shown in the Equation (2) (22, 30) “SN ∝ ∑*n*_*i*_*m*_*i*_” (*i means measurement project*). PBC refers to the individuals' perceptions of the controllability and ability to perform a given behavior (29). The two separable components to measure PBC are control beliefs (c) and perceived power (p), which are shown in the Equation (3) (22, 30) “PBC ∝ ∑*c*_*i*_*p*_*i*_ ” (*i means measurement project*). Ajzen (29) proposed that the model can also accommodate any variables that effectively explain and predict the behavior and the behavior intention when studying a particular behavior in addition to three variables: attitude, SN, and PBC. That is to say, we could add new variables to this model on a reasonable basis, which could exert an impact on the behavior belief and behavior.

**Figure 2:**
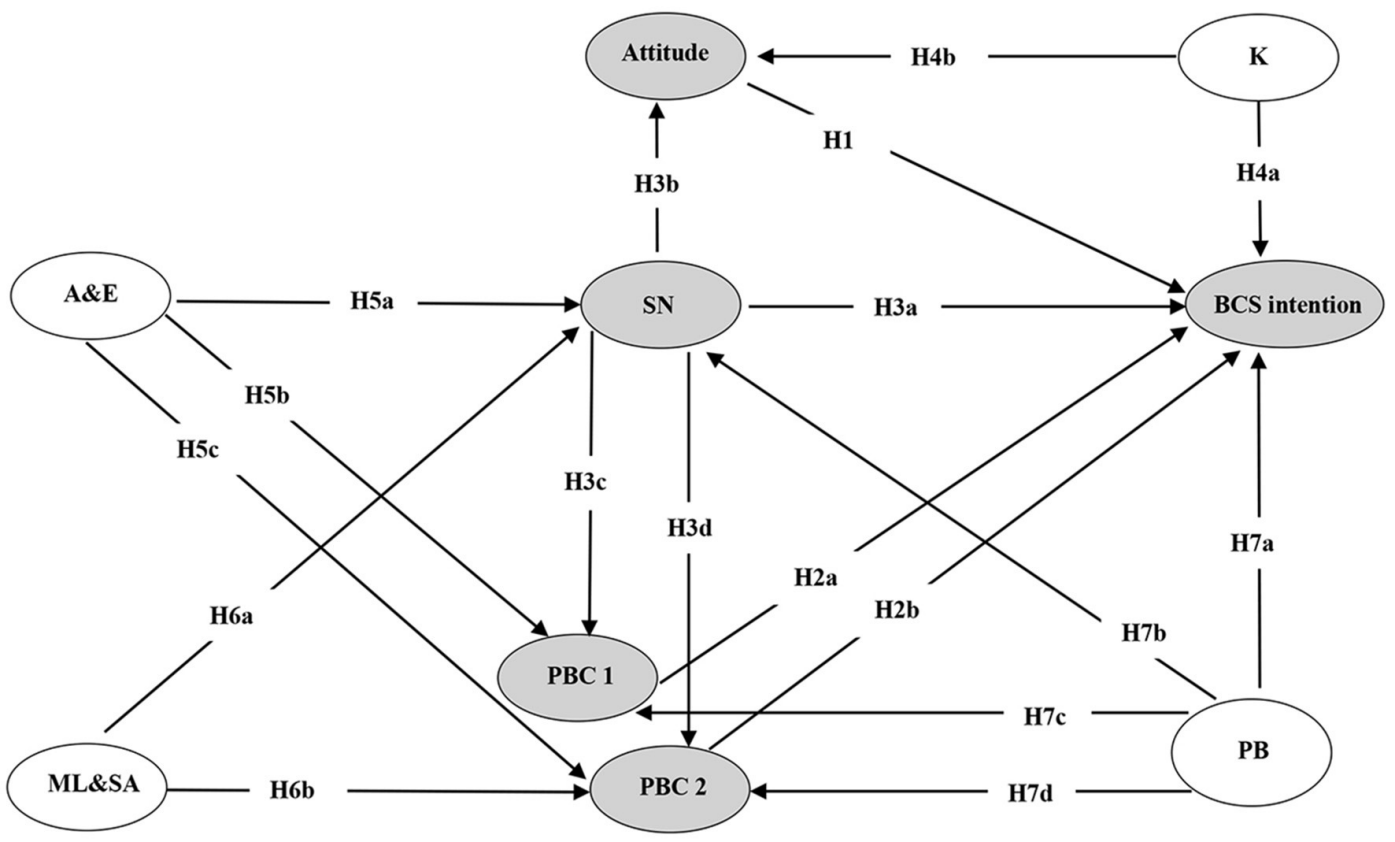
The research hypotheses and research model. SN, subjective norm; PBC 1, perceived behavioral control 1; PBC 2, perceived behavioral control 2; BCS intention, breast cancer screening intention; K, knowledge; PB, past behavior; A&E, advocacy and education; ML&SA, the medical level and service attitude. K, PB, A&E, and ML&SA are added in the model as new variables; A&E and ML&SA belong to the supply-side factors.

Combined with the existing literature, the research hypotheses and the model adopted in this study were developed based on TPB, which is shown in Figure 2.

According to TPB, the more positive the rural women's attitude is, the higher intention they will have (29). A previous study among rural women in Korea showed that lack of awareness may lead to the low participation rate in BCS tests (32). Additionally, Yan demonstrated that a negative attitude toward health check-ups was one of the reasons why female residents are less likely to be screened for BC in Macao (33). Considering this, we assumed that:

**H1: Attitude is positively associated with the rural women's BCS intention**.

Based on TPB, the rural women's discernment to be screened for BC (PBC) can directly predict the occurrence of BCS (29). Past studies demonstrated that encountered barriers, such as lack of time, long geographic distance to primary health facilities, etc., probably affect BCS (34). Therefore, the closer the distance to the township health centers or village clinics, the more convenient traffic and women's time resources, the stronger the PBC. Another stream of research revealed that it is a taboo for Asian women to show their breasts to others due to their traditional culture (35). Besides, rural women refrained from participating in BCS due to their ashamed and embarrassed reaction when exposing their breasts to male physicians (35, 36). Women also were impeded by the view that BCS is painful or uncomfortable (34). Personal fear of doctors/examiners, hospitals, and health facilities also exerted a negative impact on the women's attitudes toward BCS (19). That is to say, the less the embarrassment/fear, during the breast cancer screening, the higher the PBC score.

Based on the analysis above, we assumed that personal factors (distance, transportation, busyness, et al.) named PBC 1, pain and cultural-social factors (e.g., embarrassment) named PBC 2 both positively related to BCS for rural women.

**H2a–H2b: PBC 1 and PBC 2 are positively associated with rural women's BCS intention, respectively**.

According to TPB, SN has an effect on BI (29). If the SN varies, attitude and PBC will vary concordantly (29). It was evident that lack of encouragement from family members and physicians was one of the major inhibitors affecting women's decision on the BCS program (37). There was a significant correlation between lower social support and absence of BCS (19). Studies indicated that the social support network from women's colleagues in the workplace, families, and friends was important. Higher levels of social support networks lead to more positive attitude toward preventive health care (36, 38, 39). A study of 154 non-governmental organizations from 35 countries revealed that community health workers and local volunteers played a pivotal role in reducing women's discomfort and shyness while referring to breast health care (40). The multiple responsibilities undertaken by women in the workplace and at home, and the restriction of time urge the working women to postpone their own affairs for the sake of family members (37). Thus, it can be inferred that rural women will have more time to undertake BCS if they get more support and encouragement from their workplace or families. Based on the discussion above, we developed the following hypotheses:

**H3a: SN is positively associated with the rural women's BCS intention**.

**H3b–H3d: SN also has an effect on rural women's attitude toward BCS, PBC1, and PBC2**.

Many scholars have attempted to add new variables to the theoretical model of TPB in order to improve the explanatory power. The new variables included personality, behavior experience, anticipated regret, and so on (41). According to Ajzen's view in 1991, we also added some new variables to this model.

Initially, we added the knowledge of BC and BCS. Previous studies found that cognitive and knowledge levels affected women's intention and behavior to receive BCS services (42) or mammography screening (43). Insufficient knowledge about BC made it less likely for women to engage in BCS (33). Besides, insufficient knowledge was one of the reasons for ignoring mammography (44). Therefore, we hypothesized that:

**H4a–H4b: Knowledge has a positive effect on BCS intention and attitude**.

Advocacy and education (A&E) also play an important role in BCS. A number of research demonstrated that health education interventions have been conducted, and health education is considered one of the most important factors affecting public health (45, 46). The study also suggested that advocates for prevention could encourage women to become role models and do advocacy for screening in their communities to build positive community sentiment and shift social norms (47). Qin also mentioned that the development of community health education can reduce the rejection or concerns (48). As a result, we hypothesized that:

**H5a–H5c: Advocacy and education (A&E) have an effect on SN, PBC 1, and PBC 2**.

Medical level and service attitude (ML&SA) could influence the patients' experience and satisfaction (49, 50). The higher degree to which people satisfy with the recent medical experience, the stronger their trust in health care system will be (51). That is to say, high-level medical condition and excellent attitude could bring professional reputation and credibility; thus, women would receive more encouragement and social support from their families and friends. Additionally, patients were more likely to trust physicians who were employed by hospitals, which had better medical equipment, medical level, service, etc. (52). Therefore, we hypothesized:

**H6a-H6b: The medical level and service attitude (ML&SA) of township health centers and village clinics have an effect on SN and PBC 2, respectively**.

Previous research demonstrated that a bad experience in the past was one of the top three barriers to BCS (53). It means a bad experience may have a bad effect for rural women to be screened for BC. The study which was conducted in China (Wu et al.) suggested that past screening behavior could make women get more suggestions from health care providers, which promoted that SN plays an important role in the process of intention formation (54). Usually, a person who has a good habit or a good experience is more likely to perform the behavior and to comply with the recommendations from stakeholders than those who have not. The research also revealed that those who practiced breast self-examination monthly had a lower level of barriers than those who screened less frequently (55). Therefore, the study hypotheses are as follows:

**H7a–H7d: The past behavior experience (PB) is positively associated with BCS intention, SN, PBC1, and PBC 2**.

## Materials and Methods

### Setting

A cross-sectional study was conducted in Jiangsu province. The rural women were recruited by a convenience sampling method between July and September, 2020. In the first stage, considering their different economic development levels, we selected 3 districts from 3 regions, respectively: Lianyungang, which is located in Northern Jiangsu province; Yangzhou, which is located in Central Jiangsu; and Nanjing, which is located in the southern part of the province. In the second stage, by consulting the experts, seven survey sites in rural areas were selected, covering three districts for the present study, i.e., Donghai, Haizhou, Guanyun in Lianyungang, Gaoyou, Tangwang in Yangzhou and Qixia, Jiangning in Nanjing. In the third stage, convenience sampling was used to recruit practitioners in the seven survey sites.

### Participants and Data Collection

The participants were involved if they were women living in a rural area, more than 18 years old, and willing to participate in this research. Rural women with intellectual disability or language barrier who could not complete the questionnaires were excluded to ensure the validity of investigation. In order to improve the quality of the investigation, the questionnaire forms were filled out by a face-to-face interview with the help of trained and qualified investigators. Before obtaining answers, the investigators had explained to each participant who was required to fill out all the questions voluntarily and truthfully that the investigation was anonymous and the collected data would be only used in this study and kept completely confidential. The participants could get a bottle of laundry detergent as a reward. The price of it is 8 CNY.

The minimum sample size using PLS to measure models should not be <10 times the number of items of the most complex construct or the largest number of independent variables influencing the dependent variable (56). In the model of this study, the number of items of the most complex construct is 10. Besides, Raosoft was used to calculate the sample size as another way (57). According to the Sixth National Census in China, there were about 15.74 million rural women in Jiangsu, China (58). Therefore, the population size is estimated to be 15.74 million. The margin of error, confidence level, and the response distribution were, respectively set as 5%, 95%, and 50%. Then, the recommended sample size is 385.

### Instruments and Measures

A self-made questionnaire was designed for data collection. A pilot survey was carried out, and the questionnaire was modified properly, which made the survey more reasonable and feasible before the formal investigation. The final formal questionnaire consisted of five parts (50 items in total). Part I: attitude and views on BCS (28 items or 14 pairs in total); Part II: BCS intention and past behavior (4 items in total); Part III: the current status of township health centers or village clinics (5 items in total); Part IV: the knowledge of BC (7 items in total); Part V: the demographic characteristics of the participants (6 items in total). Especially, 28 items of attitude and views on BCS were designed to be 14 pairs, including attitude (3 pairs or 6 items), subjective norm (5 pairs or 10 items), PBC 1 (3 pairs or 6 items), and PBC 2 (3 pairs or 6 items) to measure the two separable components of these three variables, respectively, according to Ajzen's TPB questionnaire (31). The calculation equations are as follows. (1) AB ∝ ∑b_i_e_i_; (2) *SN* ∝ ∑*n*_*i*_*m*_*i*_; (3) *PBC* ∝ ∑*c*_*i*_*p*_*i*_ (*i* means the number of items measured) (22, 30).

The items of Part I, Part II, and Part III were scored on a five-point Likert scale. The scores range from 1 to 5 points. For example, women rated “saving cost of treatment” as the values 1 (“not at all important”), 2 (“not important”), 3 (“neutral”), 4 (“important”), and 5 (“very important”). Whereas, some items of PBC 1 (Item 3) and PBC 2 (Item 1, Item 3, and Item 5) were scored reversely. The items of Part IV were scored 1 if the choice were right, and 0 otherwise. The questionnaire was regarded as completed only if all the questions were answered. We substituted the mean of the respondents in the same unit for the missing data (59).

### Data Analysis and Statistics

Data were recorded using Microsoft Excel. SPSS V.22.0 was used to conduct the descriptive statistics and calculate the scores of attitude, SN, and PBC according to Equations (1–3). Considering the interrelationship on the rural women's BCS intention and the influence factors in this research model, the hypotheses were performed by the partial least square structural equation model (PLS-SEM) using Smart PLS 3.2.8. This is because it shows a minimal restriction in sample size and residual distribution, and there is no constraint on the model specification and data distribution assumptions when it is used to analyze the complex model with latent variables (60). Especially, it integrates two methods of factor analysis and path analysis, which can be used to simultaneously measure the measurement model and the structure model and estimate the factor structure and the relationship among various factors (61).

Relevant studies have found that ages were closely related to BCS (62). It is meaningful to consider the age factor when implementing and promoting the BCS program. In recent years, the rural women's upper age limitation of participating BCS program was changed from 59 to 64 years (63). The policy poses the same effects on the rural women who are below 35 years old and who are above 64 years old. Therefore, the multi-group analysis (MGA) of Group 1 (below 35 years old or above 64 years old) and Group 2 (between 35 and 64 years old) was performed to discover the differences by using Henseler's MGA and the permutation method.

### Ethics Approval

This study's ethical admission was approved by the Ethics Committee of Sir Run Run Hospital, Nanjing Medical University. The grant number is 2019-SR-017. We obtained the oral informed consent from each subject who participated in the survey.

## Results

About 3,200 questionnaires were distributed and 3,050 were returned. After removing the invalid questionnaire, 3,011 were usable. The valid response rate was 94.1%.

### Descriptive Statistics

The demographics and relevant characteristics of the interviewers are shown in Table 1. The participants in Group 1(< 35 or > 64) and Group 2 (between 35–64) of BC account for 46.3% and 53.7%, respectively. The number of participants with a secondary school degree is the highest (44.3%). The majority of the participants are married or living with a common-law partner (88.7%). The number of rural women who have access to know about the BCS (68.4%) is more than those who have not (31.6%). A total of 9 approaches to know about BCS in this survey were as follows, by decreasing frequency: doctor, nurse or health staff (*n* = 1,363), television (*n* = 989), Wechat (*n* = 935), friends or a neighbor (*n* = 707), publicity column (*n* = 600), handbooks or leaflets (*n* = 597), newspapers or magazines (*n* = 548), family members (*n* = 487), and broadcast (*n* = 309).

**Table 1:** Participant demographics and characteristics (*n* = 3,011).

**Variable**	**Values**	***N*** **(%)**
Age	Group1: <35 or >64	1,393 (46.3%)
	Group2: between 35–64	1,618 (53.7%)
Education level	Illiteracy/primary school or below	562 (18.7%)
	Junior high school/ senior high school	1,333 (44.3%)
	Collegeand above	1,116 (37.1%)
Family Income (per month)	≤ 2,000	301 (10.0%)
	2,000–5,000	1,098 (36.5%)
	5,000–10,000	953 (31.6%)
	≥10,000	659 (21.9%)
Marital status	Never married	266 (8.8%)
	Married/ cohabitation	2,670 (88.7%)
	Divorced/separated/widowed	75 (2.5%)
The ways to know about the screening	Have	2,059 (68.4%)
	Don't have	952 (31.6%)
Total		3,011 (100%)

The scores range from 1 to 5 points except the items of knowledge (1–7). Especially, according to the calculation equations of TPB, the overall scores of attitude, SN, PBC1, and PBC2 are on the scale of 1–25. As shown in Table 2, the mean score of attitude (mean score, 19.621; SD, 4.164) revealed that rural women were positive about early diagnosis and treatment (mean score, 21.027; SD, 4.965), the effect on saving cost (mean score, 19.352; SD, 5.652), and the outcome of screening (mean score, 18.485; SD, 5.011). For the construct SN, the mean score was 15.750, and the standard deviation was 4.197. The mean score of exports' effect was lowest (mean score, 12.763; SD, 6.292). The mean scores of PBC 1 (mean score, 11.809; SD, 4.062) and PBC 2 (mean score, 12.143; SD, 4.445) were not very optimistic, especially the scores of times (mean score, 9.253; SD, 4.170) and male physicians (mean score, 9.291; SD, 6.124). For A&E, the mean score was 2.967, and standard deviation was 0.886. Totally, 68.2% of rural women never/hardly/seldom received A&E on breast cancer. The mean score of ML&SA (mean score, 3.597; SD, 0.653) was also <4. As for knowledge (mean score, 4.017; SD, 2.000), 36.8% rural women scored 0–3 points. The mean score of PB was 2.394 (SD, 1.458). There were 1,256 rural women (41.7% of 3,011 participations) who were not taking part in the BCS program within the past 5 years. The mean score of rural women's behavior intention was 3.969 (SD, 0.782). There were still rural women who “strongly disagree” or “disagree” or kept “neutral” on “I plan/intend/will try to undertake BCS.” The ratio reached 27%, 19.3%, and 21.8%, respectively.

**Table 2:** Mean scores for every item (*n* = 3,011).

**Construct**	**Item**	**Scale**	**Mean ±SD**	**Construct**	**Item**	**Scale**	**Mean ±SD**
Attitude		1–25	19.621 ± 4.164	A&E		1–5	2.967 ± 0.886
	A1	1–25	21.027 ± 4.965		A&E1	1–5	2.978 ± 0.985
	A2	1–25	19.352 ± 5.652		A&E2	1–5	2.955 ± 1.015
	A3	1–25	18.485 ± 5.011	ML&SA		1–5	3.597 ± 0.653
SN		1–25	15.750 ± 4.197		ML&SA1	1–5	3.507 ± 0.743
	SN1	1–25	17.082 ± 5.205		ML&SA2	1–5	3.439 ± 0.769
	SN2	1–25	16.531 ± 5.083		ML&SA3	1–5	3.845 ± 0.730
	SN3	1–25	17.112 ± 4.965	BCS intention		1–5	3.969 ± 0.782
	SN4	1–25	15.261 ± 5.425		BI1	1–5	3.895 ± 0.871
	SN5	1–25	12.763 ± 6.292		BI2	1–5	4.031 ± 0.791
PBC 1		1–25	11.809 ± 4.062		BI3	1–5	3.981 ± 0.819
	PBC1-1	1–25	12.761 ± 5.431				
	PBC1-2	1–25	9.253 ± 4.170	Knowledge		1–7	4.017 ± 2.000
	PBC1-3	1–25	13.414 ± 5.348				
PBC 2		1–25	12.143 ± 4.445	PB		1–5	2.394 ± 1.458
	PBC2-1	1–25	11.617 ± 5.437				
	PBC2-2	1–25	9.291 ± 6.124				
	PBC2-3	1–25	15.521 ± 5.968				

*SD, standard deviation; A1–A3, denote the three paired items used to measure the respondents' attitudes; SN1–SN5, the five paired items used to measure the respondents' SN; PBC1-1–PBC1-3, the three paired items used to measure the respondents' PBC 1; PBC2-1–PBC2-3, the three paired items used to measure the respondents' PBC 2; A&E1–A&E3, the three items used to measure the respondents' views on A&E; ML&SA1-ML&SA3, the three items used to measure the respondents' views on ML&SA; BI1-BI3, the three items used to measure the respondents' BCS intention; PB, the past behavior within 5 years*.

### Evaluation of Measurement Model

As shown in Table 3, all factor loadings were significant at *p* < 0.001 on its underlying construct, showing satisfactory convergent validity. Meanwhile, Table 4 illustrated that Cronbach's α ≥ 0.600, which indicated sufficient internal consistency or reliability, and that composite reliability was adequate (64). The discriminant validity of the questionnaire was assessed. The correlation matrix for each pair of constructs is shown in Table 4. It is evident that the AVE square root of each construct is higher than the absolute value of its correlation (64); the cross-loadings show that all items loaded on their respective constructs are higher than those on the other constructs, and the cross-loadings differences are above the threshold of 0.10 (65). Finally, the HTMT ratio is below the threshold of 0.85 or 0.90 (66).

**Table 3:** Factor loadings (*n* = 3,011).

**Variables**	**Items**	**Factor loadings**	***p*** **-Value**	**Variables**	**Items**	**Factor loadings**	***p*** **-Value**
Attitude	A1	0.815	0.000	PBC 2	PBC2-1	0.821	0.000
	A2	0.773	0.000		PBC2-2	0.717	0.000
	A3	0.807	0.000		PBC2-3	0.733	0.000
SN	SN1	0.814	0.000	A&E	A&E1	0.896	0.000
	SN2	0.872	0.000		A&E2	0.875	0.000
	SN3	0.881	0.000	ML&SA	ML&SA1	0.882	0.000
	SN4	0.776	0.000		ML&SA2	0.872	0.000
	SN5	0.571	0.000		ML&SA3	0.866	0.000
PBC 1	PBC1-1	0.875	0.000	BCS intention	BI1	0.938	0.000
	PBC1-2	0.604	0.000		BI2	0.954	0.000
	PBC1-3	0.907	0.000		BI3	0.946	0.000

*A1–A3, denote the three paired items used to measure the respondents' attitudes; SN1–SN5, the five paired items used to measure the respondents' SN; PBC1-1–PBC1-3, the three paired items used to measure the respondents' PBC 1; PBC2-1–PBC2-3, the three paired items used to measure the respondents' PBC 2; A&E1–A&E3, the three items used to measure the respondents' views on A&E; ML&SA1-ML&SA3, the three items used to measure the respondents' views on ML&SA; BI1–BI3, the three items used to measure the respondents' BCS intention*.

**Table 4:** Correlations among variables (*n* = 3,011).

	**α**	**CR**	**AVE**	**Attitude**	**BI**	**ML&SA**	**PBC1**	**PBC2**	**A&E**	**SN**
Attitude	0.719	0.729	0.637	*0.798*	0.487	0.231	0.291	0.328	0.179	0.642
BI	0.941	0.942	0.895	0.408	*0.946*	0.302	0.467	0.374	0.378	0.615
ML&SA	0.846	0.906	0.763	0.184	0.270	*0.873*	0.414	0.322	0.466	0.399
PBC1	0.731	0.845	0.651	0.243	0.403	0.346	*0.807*	0.596	0.334	0.438
PBC2	0.636	0.802	0.575	0.234	0.298	0.241	0.412	*0.759*	0.180	0.397
A&E	0.725	0.879	0.784	0.129	0.310	0.365	0.265	0.117	*0.885*	0.340
SN	0.844	0.891	0.626	0.498	0.545	0.338	0.372	0.307	0.256	*0.791*

*BI, BCS intention; PBC1, perceived behavioral control 1; PBC2, perceived behavioral control 2; SN, subjective norm; ML&SA, the medical level and service attitude; A&E, advocacy and education; CR, composite reliability; AVE, average variance extracted. The square roots of the AVE are shown on the diagonal and italicized elements in gray shade, above which are the HTMT values and below which are the correlations between the construct's values*.

### Evaluation of Structural Model

**Figure 3:**
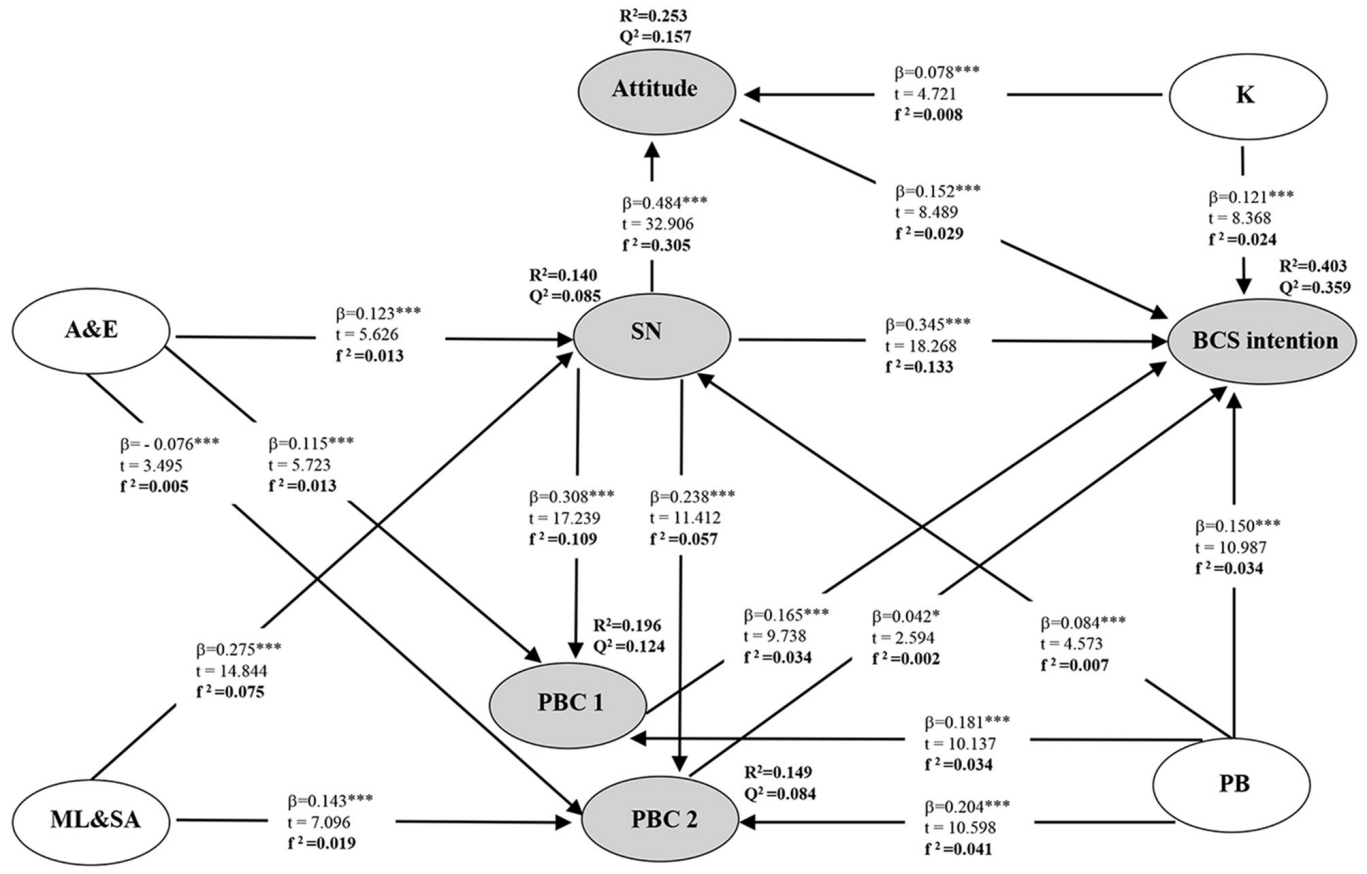
A path diagram for the research model. β, path coefficient; *** *p* < 0.001, * *p* < 0.05; *t, t*-value; R^2^, coefficient of determination, which represents the amount of explained variance of each endogenous latent variable; f ^2^, effect size; Q^2^, predictive relevance; PB, past behavior; BI, behavior intention; PBC1, perceived behavioral control 1; PBC2, perceived behavioral control 2; SN, subjective norm; K, knowledge; ML&SA, the medical level and service attitude; A&E, advocacy and education; BCS intention, breast cancer screening intention.

The model measurement results and hypothesis testing results are shown in Figure 3. About 40.3% of variance in the intention to BCS is explained: attitude is 25.3%, SN is 14%, PBC 1 is 19.6%, and PBC 2 is 14.9%. In particular, the values of f ^2^ (0.02, 0.15, and 0.35) indicate small, medium, and large effects (67). All the values of Q^2^ are considerably above zero, and this finding supports the model's predictive relevance from an out-of-sample prediction perspective (68). SRMR in this model is 0.073 (i.e., below 0.08) (69), confirming the overall fit of this PLS path model.

The path coefficient (β) and *t-*value in Figure 3 also demonstrate that all of the hypotheses are supported. Of all the factors affecting the intention to BCS, SN is found to be the strongest predictor.

Furthermore, we found some specific indirect effects in this model, as depicted in Table 5. In addition to the hypothesis put forward, we also found that SN, attitude, PBC 1, and PBC 2 all played an intermediary role in the model.

**Table 5:** Specific indirect effects (*n* = 3,011).

	**Path**	**Path coefficient (β)**	***t*** **-Value**	**p**
1	ML&SA -> SN -> A -> BI	0.02	7.132	0.000
2	K -> A -> BI	0.012	4.042	0.000
3	A&E -> SN -> A	0.059	5.596	0.000
4	A&E -> SN -> BI	0.042	5.254	0.000
5	PB -> SN -> PBC2 -> BI	0.001	2.186	0.029
6	A&E -> SN -> PBC2 -> BI	0.001	2.293	0.022
7	SN -> PBC1 -> BI	0.051	8.287	0.000
8	ML&SA -> SN -> PBC2 -> BI	0.003	2.522	0.012
9	SN -> A -> BI	0.074	8.089	0.000
10	ML&SA -> SN -> PBC1	0.085	10.202	0.000
11	A&E -> SN -> A -> BI	0.009	4.657	0.000
12	PB -> PBC1 -> BI	0.03	6.995	0.000
13	ML&SA -> SN -> PBC2	0.065	9.182	0.000
14	PB -> SN -> BI	0.029	4.483	0.000
15	A&E -> PBC2 -> BI	−0.003	2.132	0.033
16	ML&SA -> PBC2 -> BI	0.006	2.419	0.016
17	ML&SA -> SN -> A	0.133	13.062	0.000
18	ML&SA -> SN -> PBC1 -> BI	0.014	6.905	0.000
19	PB -> SN -> PBC1 -> BI	0.004	4.031	0.000
20	ML&SA -> SN -> BI	0.095	11.251	0.000
21	PB -> SN -> PBC1	0.026	4.424	0.000
22	PB -> PBC2 -> BI	0.009	2.517	0.012
23	PB -> SN -> PBC2	0.02	4.221	0.000
24	A&E -> SN -> PBC1 -> BI	0.006	4.675	0.000
25	SN -> PBC2 -> BI	0.01	2.556	0.011
26	A&E -> SN -> PBC2	0.029	5.073	0.000
27	A&E -> SN -> PBC1	0.038	5.406	0.000
28	PB -> SN -> A	0.041	4.52	0.000
29	PB -> SN -> A -> BI	0.006	3.965	0.000
30	A&E -> PBC1 -> BI	0.019	4.804	0.000

*PB, past behavior; BI, BCS intention; PBC1, perceived behavioral control 1; PBC2, perceived behavioral control 2; SN, subjective norm; A, attitude; K, knowledge; ML&SA, the medical level and service attitude; A&E, advocacy and education*.

Meanwhile, we assessed the group difference in the multi-group analysis (MGA) of age in Table 6. Both Henseler's MGA and permutation method confirmed the significance or non-significance of the differences in all results, which strengthened the findings of this research. The output of MAG reveals that there are significant differences between the two age groups in regard to the effect of A&E on PBC2 (H5c) (*p* < 0.01) and PB on BI (H7a) (*p* < 0.001). However, there is no difference in other hypotheses according to the GMA results. In the group of women whose age are between 35 and 64, PBC 2 have a positively effect on BI (H2b) (*p* < 0.05), while there is no significant influence of H2b in the group of women whose age is below 35 or above 64 (*p* > 0.05). Similar results of H7b within these two groups are also obtained.

**Table 6:** Assessment of group difference in age (*n* = 3,011).

**Hypotheses**	**Path coefficient (β)**		***t*** **-Value**		**Path coefficient differences**	**p**		**Supported**
	**Group 1:** **<35/>64**	**Group 2:** **between** **35–64**	**Group 1:** **<35/>64**	**Group 2:** **between** **35–64**		**Henseler MGA**	**Permutation**	
H1	0.177^***^	0.128^***^	6.641	5.244	0.049	0.174	0.189	No/No
H2a	0.172^***^	0.164^***^	6.879	7.187	0.008	0.821	0.833	No/No
H2b	0.051	0.045^*^	1.955	2.211	0.005	0.876	0.873	No/No
H3a	0.354^***^	0.334^***^	13.204	12.577	0.02	0.606	0.612	No/No
H3b	0.461^***^	0.51^***^	21.298	27.307	−0.049	0.086	0.097	No/No
H3c	0.296^***^	0.331^***^	11.678	13.129	−0.035	0.333	0.336	No/No
H3d	0.23^***^	0.271^***^	7.159	10.19	−0.041	0.321	0.324	No/No
H4a	0.094^***^	0.125^***^	4.151	6.695	−0.03	0.305	0.296	No/No
H4b	0.091^***^	0.061^**^	3.842	2.776	0.031	0.341	0.358	No/No
H5a	0.130^***^	0.114^***^	4.182	3.830	0.016	0.712	0.732	No/No
H5b	0.155^***^	0.089^**^	5.198	3.225	0.066	0.106	0.108	No/No
H5c	0.005^**^	−0.127^***^	0.158	4.338	0.132	0.002	0.003	Yes/Yes
H6a	0.271^***^	0.277^***^	9.317	11.591	−0.006	0.874	0.865	No/No
H6b	0.127^***^	0.127^***^	4.183	4.684	0.000	0.997	0.996	No/No
H7a	0.097^***^	0.200^***^	4.704	10.857	−0.103	0.000	0.000	Yes/Yes
H7b	0.051	0.119^***^	1.950	4.760	−0.068	0.06	0.073	No/No
H7c	0.141^***^	0.134^***^	5.358	5.435	0.007	0.852	0.846	No/No
H7d	0.181^***^	0.147^***^	6.075	5.905	0.033	0.388	0.387	No/No

*** *p < 0.001*,

** *p < 0.01*,

* *p < 0.05*.

## Discussion

In this study, we added four exogenous variables to the TPB model, such as knowledge, past behavior, and supply-side factors (A&E and ML&SA). The data are well in accordance with the theoretical predications. Firstly, the addition of variables strengthened the explanatory power of the TPB model and further demonstrated the utility of TPB for prediction. Secondly, health policy makers and interveners could get more information in the decision-making and in the intervention process and improve the intervention effect. Finally, extending variables into the TPB model, the findings in this paper demonstrate that the original TPB model can be further developed and applicable to other areas, particularly to those related to public health.

Although BC has the highest cancer incidence rate in the world and BCS can prevent it effectively, most rural women in China, even in the economically well-developed area, had a lower willingness to be screened for BC (13–15). Our study supported this result. Our findings indicated that it is significant to explore the influencing factors of BI to BCS in rural China, especially the differences between the Group 1 and Group 2.

This study revealed how various psychosocial factors, including attitude, SN, PBC, and other external factors, such as knowledge, past behavior, and supplier factors (A&E and ML&SA), impact on BCS intention. It also provided evidence that TPB could well-explain and predict rural women's BCS intention. As hypotheses, attitude, SN, PBC 1, PBC 2, knowledge, and PB were positively related to the BCS intention, and they also played an intermediary role between the relationship of A&E and BI, ML&SA and BI. Besides, in the MGA of age, we found some significant differences between the two groups.

The finding of this research confirmed the positive relationship between attitude and rural women's BSC intention, which is consistent with the results of the similar studies on rural women in Korea (32) and female residents in Macao (33). Attitude also links other variables. Therefore, in order to improve willingness, we should constantly improve the attitude of rural women toward BCS. It cannot be overemphasized that BCS is beneficial for the early diagnosis and treatment of cancer. Therefore, health institutions can provide lectures on successful cases to rural women to make them realize the importance of screening and early diagnosis. Meanwhile, the government could give the preferential policies to conduct free screening for age-appropriate women, which can improve the attitude toward BCS and further enhance the behavioral intention.

In line with the results of Saudi (34), Korea (35), and Hong Kong (36), lack of time, long geographic distance, painful and uncomfortable experience during the examination, and other factors are obstacles that rural women encountered. This result can be possibly ascribed to the limited detection and screening facilities in some areas (5). It is common for rural women in China to hold a relatively conservative attitude toward their bodies. Shang's research held the opinion that examining bodies by oneself or by others was regarded as inappropriate behavior (70). These conservative social norms may help explain why Chinese rural women's feelings of embarrassment or shyness become the key barriers to be screened for BC in this study, and the result is also consistent with the past results of Im's (35). As a result, rural women must overcome some difficulties when they participate in BCS. Besides, the same thing as attitude is that PBC also is linked to other variables. Therefore, in order to improve women's BCS intention, we need to reduce the hindering factors and facilitate the promoting factors of BCS intention in rural women. Firstly, rural women could be organized to go to the hospitals or clinics for BCS. It is, maybe, a good way to implement an appointment system to reduce transportation and time costs. Next, in order to decrease bad feelings, we could show the screening process and the use of equipment in the form of an animated short film to improve rural women's understanding. Besides, it may be useful to increase the number of female physicians in order to reduce the embarrassment of being examined. Furthermore, we could conduct psychological counseling on embarrassment and fear.

At the same time, when designing various interventions to reduce embarrassment and fear and to increase a BCS rate, ML&SA, which is a positive influence factor of PBC, is closely related to the patient feelings and also needs to be considered. In this study, ML&SA can positively affect SN and PBC 2, and it can also influence BI through attitude, SN, and PBC 1. The following suggestions could be referred: (I) The health authority should make a regular screening training for physicians in the health center to improve their screening ability. The better the screening level of medical staff is, the fewer feelings of fear and pain. (II) The government could cooperate with social institutions to increase the funding of primary healthcare infrastructure. They should further improve the software and hardware and promote the upgrading of village clinics' screening facilities. (III) All healthcare physicians should respect and protect women's privacy during BCS. In turn, the rural women will show less embarrassment.

Mass media, relatives, friends, and healthcare providers are the main primary information sources in China (71), and are the widely used approaches for rural women to know about BCS in this study. The results of this study show that A&E, one of the supply-side factors, has positive effects on SN and PBC 1, which means that A&E plays an important role in obtaining social support and reducing the obstacles of the distance, transportation, busyness, etc. Furthermore, it can affect BI through attitude, SN, PBC 1, and PBC 2. However, 68.2% of rural women selected “never” or “hardly” or “seldom” with regard to the item “How often do you receive A&E on breast cancer.” A systematic review of cancer screening interventions among Asian women had the view that it was ineffective to perform the print materials and media campaigns alone (72). Therefore, given our research findings, more intervention approaches should be taken to improve the efficacy of A&E, such as television, WeChat, publicity columns, brochures or leaflets, newspapers or magazines, and broadcast. This result also shows that A&E has negative effects on PBC 2, and this is not exactly unexpected. A&E might publicize the harm of breast cancer and increase rural women's screening intention, but it could also increase the exposure of the screening process. Thus, they may feel more embarrassed, especially with the male physician's involvement. Hence, A&E should provide positive psychological support and improve the education system. Privacy protection deserves a special attention. Besides, in this study, 66.7% of rural women “never” or “hardly” or “seldom” are advised to participate in the BCS by the physicians, which demonstrates again that healthcare physicians do not play a crucial part in the A&E. Considering that, it is necessary to collaborate with SN, e.g., healthcare providers, healthcare physicians, relatives or friends, to expand the influence of A&E.

In this study, SN is the strongest predictor of rural women's screening intention. SN is positively related to the rural women's BCS intention, which is similar to the existing research results of Parsa (37) and Jensen (19). Besides, it also has positive effects on rural women's attitude toward BCS, PBC 1, and PBC 2, which are consistent with the TPB model and similar to previous studies (36, 38, 39). Hence, it is necessary for the government to encourage the stakeholders to fully support rural women to conduct BCS. Family members and good friends should give psychological comfort and support to reduce the obstruction of PBC. Primary care physicians in the clinic and experts could introduce the relevant knowledge and importance of BCS to rural women. Beyond that, as a hub in the research model, SN combines other influencing factors (e.g., A&E, ML&SA, PB) and plays an intermediary role. Therefore, it is useful that rural women who have been screened in the past talk about their experience in the A&E program. For rural women who have never participated in screening, it is also beneficial for them to be familiar with the screening process. These ways can make rural women aware of the necessity for screening and follow the doctor's advice for timely screening.

The result in this study shows that having sufficient knowledge about breast cancer has a positive effect on rural women's BCS intention and attitudes toward BCS, which is consistent with the studies of Ana (44), Coyne (42), Berry (43), and Yan (33). Knowledge also influences BCS intention through attitude. The past research reported that lack of knowledge may prevent women from identifying the main symptoms of the disease and consequently lead to the neglect of the disease, which can result in a delay of detection (5, 73). Moreover, 44% of participants who get a score of 0 in the item “Which preventive measures can early detect breast cancer lesions.” This result indicates that rural women's knowledge is insufficient and the prevention awareness of BC is unsatisfying. The relevant study also revealed that poor knowledge about BCS contributes to a negative attitude (54). In a certain sense, many rural women do not believe that they are at risk of BC. Authorities should strengthen the popularization of knowledge about BCS, such as lectures and videos, to help rural women learn breast examination methods. We should also encourage rural women to accept regular physical examination, including breast self-examination and physical diagnosis by physicians or professional nurses every year. Breast examination of different age groups should be taken additionally.

Past behavior is also positively associated with BCS intention. We also found that PB has a positive effect on SN, PBC 1, and PBC2 directly. It also indirectly affects BCS intention through SN, PBC 1, and PBC 2. A recent review has reported that women who have been screened for BC have more opportunities to get suggestions about the prevention of breast cancer from the physicians (54). Enhanced communication between doctors and rural women can encourage rural women to follow the doctor's advice and get screened. Rural women who have done screening are also better aware of the screening process, which can reduce their fear. This study reported that 41.7% of 3,011 participations were not taking part in the BCS program within the past 5 years. As a result, we should give more encouragement to those rural women who had never been screened for BC.

The output of MAG reveals that there are significant differences between Group 1 (<35 or >64) and Group 2 (between 35–64) in regard to the relationship between A&E and PBC2. Especially, the effects of both groups are significant, but signs of the path coefficients are opposite: “+” for Group 1; “-” for Group 2. We figured out a possible reason. As A&E about BC-free screening program mainly targeted on rural women between 35 and 64 years old, they believe that A&E might lead to more and more people knowing that they will attend the screening, which would make them apprehensive and embarrassed. The MGA results also reveal that there is a significant difference between the two groups in the effect of PB on BCS intention, and the path coefficient of Group 2 is higher than that of Group 1. Besides, PB has a positive effect on SN for the rural women who are in Group 2, but it has no significant impact for the rural women who are in the Group 1. According to the results of MGA, in Group 2, PBC 2 has a positive effect on BI, despite little significant influence in the other group. It can be deduced that the respondents who were in Group 2 were more sensitive than those women who were in Group 1 on embarrassment, fear, and pain. In summary, the rural women in Group 2 were more susceptible to the practical behavior and practical experience from themselves or primary care physicians, while those rural women in Group 1 were more susceptible to advocacy and education. Therefore, the differences in the different stages of age could be considered in designing policy inventions. We should pay special attention to Group 2. For instance, township health centers and village clinics should schedule more female physicians and improve the healthcare physicians' screening experience. Medical institutions should publicize cancer screening among rural women through lectures or other ways for the rural women in Group 1, while, for the rural women in Group 2, the past experience and behavior of participating in screening should be emphasized, and their privacy should also be protected.

In this study, by multi-stage stratified sample method, we are concerned with the influencing factors of BCS intention of these women who are living in rural areas with different economic development levels in Jiangsu, China. Therefore, the participants of this study were representatives of rural women groups. Considering differences in economic development levels, our study results can be generalized to other rural areas across China. We have to acknowledge that there are still some limitations to this study. First, this study collected information in the form of a self-filled questionnaire. Therefore, an inaccurate estimation of BCS and recall bias were unavoidable. Second, our data came from a sample of rural women of some areas in Jiangsu Province, limiting generalizability to the urban area. Third, we could not judge causal inferences between TPB factors and actual screening behavior due to the cross-sectional study method, which did not control all possible confounding variables. Future studies should test the causal relationship by a research design of prospective control.

## Conclusion

In this study, we investigated the influencing factors of Chinese rural women's BCS intention by a PLS-SEM approach based on TPB and proposed some intervention measures. Among all the factors affecting the intention to BCS, SN is found to be the strongest predictor, followed by PBC 1, attitude, PB, knowledge, and PBC 2. A&E and ML&SA can affect BI through attitude, SN, and PBC. The results of MGA of age indicated that there are significant differences in different path coefficients. The findings of this study provided a theoretical basis for the implementation of intervention measures to enhance rural women's BCS willingness, which is of great significance to improve rural women's health levels.

## Data Availability Statement

The original contributions presented in the study are included in the article/supplementary materials, further inquiries can be directed to the corresponding author.

## Ethics Statement

This study's ethical admission was approved by the Ethics Committee of Sir Run Run Hospital, Nanjing Medical University. The grant number is 2019-SR-017. We obtained the oral informed consent from each subject who participated in the survey.

## Author Contributions

YH and YS: methodology, software, and writing—review and editing. YH, YS, and WL: writing—original draft preparation. YS, JY, WL, BQ, ZH, JL, and YH: investigation and data curation. YS and JY: visualization. YH: conceptualization, resources, supervision, project administration, and funding acquisition. All authors contributed to the article and approved the submitted version.

## Funding

This study was supported by the National Natural Science Foundation of China (Grant No. 71804074) and the China Medical Board (Grant No. 17-277). The funders had no role in study design, data collection and analysis, decision to publish, or preparation of the manuscript.

## Conflict of Interest

The authors declare that the research was conducted in the absence of any commercial or financial relationships that could be construed as a potential conflict of interest.

## Publisher's Note

All claims expressed in this article are solely those of the authors and do not necessarily represent those of their affiliated organizations, or those of the publisher, the editors and the reviewers. Any product that may be evaluated in this article, or claim that may be made by its manufacturer, is not guaranteed or endorsed by the publisher.

## Abbreviations

PB: past behavior
A&E: advocacy and education
ML&SA: medical level and service attitude.
